# Insecure Minds through the Looking Glass: The Mediating Role of Mentalization in the Relationships between Adult Attachment Styles and Problematic Social Media Use

**DOI:** 10.3390/ijerph21030255

**Published:** 2024-02-22

**Authors:** Gianluca Santoro, Antonino Costanzo, Christian Franceschini, Vittorio Lenzo, Alessandro Musetti, Adriano Schimmenti

**Affiliations:** 1 Department of Humanities, Social Sciences and Cultural Industries, University of Parma, Borgo Carissimi 10, 43121 Parma, Italy; alessandro.musetti@unipr.it; 2 Department of Human and Social Sciences, UKE—Kore University of Enna, Piazza dell’Università, 94100 Enna, Italy; costanzo.antonino@unikore.it (A.C.); adriano.schimmenti@unikore.it (A.S.); 3 Department of Medicine and Surgery, University of Parma, Via Volturno 39, 43125 Parma, Italy; christian.franceschini@unipr.it; 4 Department of Educational Sciences, University of Catania, Via Biblioteca 4, 95124 Catania, Italy; vittorio.lenzo@unict.it

**Keywords:** problematic social media use, attachment, mentalization, mediation model

## Abstract

Research shows that insecure attachment styles and failures in mentalizing are associated with increased problematic social media use (PSMU). This study aimed to investigate the mediating role of failures in mentalizing in the relationships between attachment styles and PSMU within a large sample of individuals from the community. The study involved the participation of 3600 adult volunteers (2312 females, 64.2%) aged between 18 and 60 years old (M = 29.92; SD = 10.68). Participants completed measures to assess socio-demographics, adult attachment styles, mentalization, and PSMU. Findings showed that secure and dismissing attachment styles predicted reduced levels of PSMU, and that preoccupied and fearful attachment styles predicted increased levels of PSMU. The relationships between adult attachment styles and PSMU were mediated by failures in mentalizing. Thus, individuals with preoccupied and fearful attachment styles may excessively resort to social media as a means of coping with unprocessed mental states. Clinical interventions that focus on improving mentalizing abilities and promoting the adoption of appropriate self-regulation strategies might reduce maladaptive engagement in social media.

## 1. Introduction

Social media encompasses a variety of digital platforms that enable individuals to share information, interact with others, and participate in communities. As of 2022, there were over 4.59 billion social media users worldwide, and this number is expected to rise to nearly six billion by 2027 [1]. Research shows that the use of social media may have both favorable and unfavorable consequences on individuals’ well-being, depending on their psychological differences [2,3,4].

In recent decades, there were suggestions that an excessive involvement with Internet platforms, such as social media, could be conceptualized as a behavioral addiction that shares similar characteristics with substance or gambling addictions [5,6,7]. For example, the components model of addiction posits that all forms of addiction are characterized by six core symptoms, which are salience, tolerance, mood modification, relapse, withdrawal, and conflict [6]. Although there is agreement about the potential for addictive behaviors related to online activities, some scholars proposed that the problematic use of Internet platforms can be understood as a compensatory strategy aimed at coping with personal and interpersonal vulnerabilities [8,9,10]. This approach focuses on the psychological processes underlying the problematic use of Internet platforms and may play a significant role in reducing the risk of overpathologizing temporary dysfunctional patterns of online activities [11]. In this study, we will use the term “problematic social media use” (PSMU) to broadly refer to excessive or otherwise dysregulated use of social media, such that it leads to addictive-like symptoms (e.g., mood modification and relapse) and impairments in various areas of individual functioning, including health, work, intimate relationships, and so forth [12,13]. Notably, previous research shows that the problematic use of Internet platforms might reflect a spectrum that encompasses different problematic online behaviors, including excessive gaming, cyberchondria, problematic cybersex, dysregulated online shopping, problematic online gambling, and PSMU [14]. Accordingly, each type of problematic online behavior may exhibit distinct characteristics and be linked to specific risk factors [15]. Hence, it is imperative to account for individual motivations and pertinent psychological mechanisms that may underpin the misuse of specific Internet platforms, along with the persistence over time and clinical relevance of the maladaptive behaviors on the Internet [16].

In this respect, research supports the role of PSMU in coping with psychological and interpersonal difficulties, also showing that PSMU is associated with several psychological vulnerabilities, such as emotion dysregulation [17,18,19], alexithymia [20,21], fear of missing out [22,23,24], low self-esteem [25,26,27], and loneliness [28,29].

### 1.1. Adult Attachment Styles and PSMU

Adult attachment styles may play a relevant role in PSMU [30,31]. Attachment is an innate motivational system that prompts individuals to form intimate relationships with others throughout their lifespan, aiming to attain a sense of safety and comfort [32,33]. Experiences in early attachment relationships shape the representations of the self, others, and the relationship between the self and others—also known as internal working models—which in turn have a profound impact on the individual’s cognitive, affective, and social development [34]. It is noteworthy that individuals might display secure, anxious, or avoidant attachment attitudes in close relationships during adulthood. Individuals with secure attachment attitudes are prone to perceiving others as available and being self-confident in close relationships. Individuals with anxious attachment attitudes tend to perceive themselves as unworthy, experience a fear of rejection, and depend on others. Individuals with avoidant attachment attitudes are prone to self-reliance and tend to devalue intimacy in close relationships [35]. Bartholomew and Horowitz [36] divided adult attachment into four categories, namely secure, dismissing, preoccupied, and fearful attachment styles, by referring to positive and/or negative representations of the self and others: secure attachment style is characterized by a positive representation of both self and others; dismissing attachment style is characterized by a positive representation of self and a negative representation of others; preoccupied attachment style is characterized by a negative representation of self and a positive representation of others; and fearful attachment style is characterized by a negative representation of both self and others. Additionally, adult attachment styles can be differentiated based on the levels of attachment anxiety and avoidance in close relationships. High levels of attachment anxiety are underpinned by a negative representation of the self, while elevated attachment avoidance is associated with a negative representation of others.

A recent systematic review showed different patterns of association between attachment and PSMU depending on the users’ developmental stage and their interpersonal context [31]. For example, secure attachment to the mother, rather than the father, was found to be negatively associated with PSMU among adolescents [37,38], and insecure attachment to peers was found to be associated with heightened levels of PSMU among older but not younger adolescents [39]. Additionally, existing literature supports the relevant role of attachment across various relational contexts in PSMU, suggesting that secure attachment might prevent the onset of PSMU, and that anxious attachment attitudes might foster PSMU [31]. Individuals with secure attachment attitudes may employ social media to strengthen the bonds within close relationships [40,41], whereas individuals with anxious attachment attitudes might engage in PSMU to satisfy their needs for relatedness and self-presentation, concurrently alleviating their fear of rejection [42,43]. However, there are inconsistent findings concerning the relationship between avoidant attachment attitudes and PSMU [31]. For example, Worsley and colleagues [44] investigated the mediating effects of attachment attitudes and depressive symptoms on the relationship between childhood maltreatment and PSMU among young adults. Their findings show that anxious attachment attitudes were associated with increased levels of PSMU, whereas avoidant attachment attitudes were associated with decreased levels of PSMU. Similarly, Liu and Ma [45] examined the potential role of emotion regulation as a mediating variable in the association between insecure attachment attitudes and PSMU within a sample of college students. They found that anxious attachment attitudes were associated with increased levels of PSMU, and that emotion dysregulation has indirect effects on this relationship. However, avoidant attachment attitudes were not significantly associated with PSMU. In contrast, Monacis and colleagues [46] investigated the associations between attachment attitudes and PSMU among adolescents and young adults. They found that PSMU was predicted negatively by confidence and discomfort with closeness—which are core characteristics of secure and avoidant attachment attitudes, respectively—and positively by the need for approval and relationship as secondary—which are core characteristics of anxious and avoidant attachment attitudes, respectively.

Additionally, research shows that preoccupied and fearful attachment styles—characterized by high levels of anxiety embedded in a negative view of the self—are associated with increased levels of PSMU, and that other psychological factors might contribute to explain these relationships. Accordingly, Costanzo and colleagues [47] found that the tendency to excessively resort on fantasy (i.e., maladaptive daydreaming [48]) partially mediated the relationship between preoccupied attachment style and PSMU, and fully mediated the relationship between fearful attachment style and PSMU. Gori and colleagues [49] showed that preoccupied and fearful attachment styles were associated with increased levels of PSMU, and that these relationships were fully mediated by low self-esteem, fear of missing out, and a larger amount of time spent on social media. It is advisable that further research identifies additional psychological factors involved in the relationship between attachment styles and PSMU to enhance understanding of the processes that might foster PSMU in individuals with an insecure attachment style, and to assist clinicians in planning tailored interventions.

### 1.2. Adult Attachment Styles, Failures in Mentalizing and PSMU

Mentalizing activities might hold a relevant influence on PSMU. Mentalization (or “reflective functioning”) is the ability to represent one’s own and other’s behaviors in terms of mental states, and is highly involved in emotion regulation [50,51]. The development of mentalization is embedded in early attachment relationships. In fact, parental reflective functioning—that is, the caregivers’ capacity to represent both their own and their offspring’s mental states [52,53,54]—plays a critical role in understanding and satisfying child’s needs, enhancing emotion regulation abilities and attachment security [55,56,57]. Conversely, child abuse and neglect are associated with insecurity in close relationships and impairments in mentalizing abilities [58,59,60].

Adult attachment styles are associated with different emotion regulation strategies in response to distressing events, potentially influencing mentalizing abilities. Individuals with a secure attachment style perceive others as available and, thus, are prone to seek comfort and assistance within close relationships. Individuals with anxious attachment attitudes, who have a negative view of themselves, tend to resort on the hyperactivation of the attachment system, exhibiting intense attempts to attain the proximity of others. Finally, individuals with avoidant attachment attitudes, thus displaying a negative view of others, are prone to resort to the deactivation of the attachment system, implying avoidance of intimacy and self-reliance [35]. Secure attachment strategies are associated with adequate mentalizing abilities during distressing events, whereas hyperactivating and deactivating strategies might increase the risk of failures in mentalizing [61]. However, the employment of deactivating strategies may enable individuals to exclude distressing feelings associated with negative attachment experiences from awareness while still retaining the capacity to cognitively process their own and others’ mental states, given that distress levels are not excessively elevated [33]. There is consistent evidence showing that insecure attachment, specifically preoccupied and fearful attachment styles, are associated with increased failures in mentalizing [62,63,64,65,66].

Research suggests that failures in mentalizing are involved in addictive behaviors [67,68,69,70], including problematic online behaviors such as PSMU [71,72,73]. In fact, difficulties in identifying and regulating distressing feelings may increase the risk of resorting to substance use or engaging in dysfunctional behaviors as a means of seeking relief [74,75,76]. Accordingly, failures in mentalizing one’s own and others’ mental states might lead to the development and maintenance of PSMU as a coping strategy to deal with unprocessed feelings and to avoid the perceived unpredictability of face-to-face interactions [72,73]. Bassi and colleagues [77] examined the relationships between childhood maltreatment, anxious and avoidance attachment attitudes, failures in mentalizing, and PSMU symptoms in a sample of emerging adults. Results show that failures in mentalizing were directly associated with increased levels of childhood maltreatment, anxious attachment attitudes, and PSMU symptoms. Thus, individuals who were exposed to child maltreatment and display anxious attachment attitudes are prone to experiencing failures in mentalizing, which in turn fosters PSMU. However, no studies were conducted to investigate the potential mediating effects of failures in mentalizing on the associations between adult attachment styles (i.e., secure, dismissing, preoccupied, and fearful) and PSMU.

### 1.3. Aims of the Study

In light of current literature, this study aimed to test the mediating role of failures in mentalizing in the relationships between adult attachment styles and PSMU. In fact, individuals with a secure attachment style might maintain adequate mentalizing abilities in response to distressing events, displaying a lower risk of engaging in PSMU. Accordingly, secure attachment styles could be negatively associated with levels of PSMU, and reduced failures in mentalizing could mediate this relationship. In contrast, individuals with preoccupied or fearful attachment styles might excessively resort on social media in order to cope with unprocessed feelings by seeking emotional support from others. Thus, failures in mentalizing could mediate the positive association between preoccupied and PSMU, and between fearful attachment and PSMU. Finally, individuals with a dismissing attachment style might display a lower risk for impairments in cognitively representing one’s own and others’ inner experiences, and avoid to engaging in interactions through social media use. Therefore, it is expected that decreased levels of failures in mentalizing mediate the negative association between dismissing attachment style and PSMU.

Additionally, there is some evidence that being female, a younger age, having lower levels of education, and a greater amount of time spent on social media are associated with increased levels of PSMU [78,79,80,81,82,83]. Accordingly, we investigated the effects of sociodemographic variables and self-reported time spent on social media in the relationships between adult attachment styles, failures in mentalizing, and PSMU.

## 2. Materials and Methods

### 2.1. Participants and Procedures

The sample of the study consisted of 3600 adult individuals (2312 females, 64.2%). The age range of the participants was between 18 and 60 years (M = 29.92; SD = 10.68). On average, participants completed 14.75 years of education (SD = 2.70). Participants were recruited by means of advertisements that were posted on social media platforms, such as Facebook and WhatsApp. Each advertisement included a link to an online survey, which comprised an informed consent schedule, a sociodemographic schedule, and different self-report measures. Those individuals who electronically signed the informed consent were automatically directed to complete the sociodemographic schedule and self-report measures. All the participants entered the study willingly and no compensation was provided to them. The current study was carried out as a component of a broader research project, which obtained approval from the Ethics Committee of the Center for Research and Psychological Intervention (CERIP) of the University of Messina (protocol code: 119094). A previous, reduced version of the dataset was employed to perform a study on the relationship between child maltreatment, adult attachment, failures in mentalizing, and PSMU in N = 1614 emerging adults (807 females, 50%) aged between 18 and 30 years old (M = 23.84; SD = 3.21) [77]. The study was conducted in adherence to the Helsinki Declaration.

### 2.2. Measures

The Bergen Social Media Addiction Scale (BSMAS; [84,85,86]) is a self-report measure that assesses social media addiction in accordance with the component model [6]. The BSMAS includes six items rated on a 5-point Likert scale (1 = “very rarely”; 5 = “very often”). The following question is an example of an item: “How often during the last year have you used social media so much that it has had a negative impact on your job/studies?”. Research supported the validity and reliability of the BSMAS [87,88,89]. However, recent evidence suggests that some items of the BSMAS might fail to assess social media addiction as a unitary construct, and thus, to differentiate excessive engagement in social media from social media addiction [12]. Accordingly, in the current study, we used the BSMAS total score to evaluate PSMU. Cronbach’s alpha was 0.83 for the full scale.

The Relationship Questionnaire (RQ; [36,90]) is a self-report measure that assesses adult attachment styles, including secure, dismissing, preoccupied, and fearful, through four first-person statements. Participants were asked to indicate how each statement represented them on a 7-point Likert scale (1 = “strongly disagree”; 7 = “strongly agree”). The following statement is an example of an item: “I want to be completely emotionally intimate with others, but I often find that others are reluctant to get as close as I would like. I am uncomfortable being without close relationships, but I sometimes worry that others don’t value me as much as I value them” (referred to the preoccupied attachment style). The RQ showed discriminant validity [91] and test-retest reliability [92].

The Reflective Functioning Questionnaire (RFQ; [93,94]) is a self-report instrument that assesses mentalizing capacities. It consists of eight items rated on a 7-point Likert scale (1 = “strongly disagree”; 7 = “strongly agree”). The following first-person statement is an example of an item: “If I feel insecure I can behave in ways that put others’ backs up”. The RFQ includes two six-item subscales, namely certainty and uncertainty about mental states. The Italian version of the RFQ subscales demonstrated satisfactory psychometric properties, which include adequate factor structure, construct validity, and reliability [94]. In the current study, we used the uncertainty about mental states subscale to evaluate failures in mentalizing. The Cronbach’s alpha of this subscale was 0.63.

A sociodemographic schedule was administered to collect information on sex, age, years of education, and the daily average amount of hours spent on social media.

### 2.3. Statistical Analyses

Descriptive statistics were computed for all variables. Sex differences for age, years of education, self-reported time spent on social media, adult attachment styles, failures in mentalizing, and PSMU were examined through *t*-test. Pearson’s *r* correlation coefficients were computed to investigate the associations between age, years of education, self-reported time spent on social media, adult attachment styles, failures in mentalizing, and PSMU. A multiple linear regression analysis was performed to test whether adult attachment styles and failures in mentalizing predicted PSMU, taking into account the effects of sociodemographic characteristics (i.e., sex, age, and years of education) and self-reported time spent on social media. Finally, four mediation analyses were computed to investigate the mediating effects of failures in mentalizing in the relationships between adult attachment styles and PSMU. Thus, each model included one of the adult attachment styles (i.e., secure, dismissing, preoccupied, and fearful) as an independent variable, failures in mentalizing as a mediator variable, and PSMU as the dependent variable. The other adult attachment styles, sociodemographic characteristics, and self-reported time spent on social media were inserted as covariates in the models. Scores on independent and mediator variables were mean-centered in order to reduce collinearity. The significance of indirect effects was tested by performing 5000 percentile bootstrap samples. The critical level for determining statistical significance was established by setting a *p*-value of 0.05: the indirect effect of the mediation model can be considered significant if the value of 0 is not encompassed within the 95% confidence interval. Mediation analyses were computed using Process Macro version 4.1 for SPSS [95].

## 3. Results

### 3.1. Descriptive Statistics and Sex Differences

Descriptive statistics and sex differences are reported in Table 1. The mean scores on the scales evaluating secure, dismissing, preoccupied, and fearful attachment styles (ranging from 2.52 to 3.31) suggest a heterogeneous distribution of adult attachment styles among participants. Additionally, the mean scores on the scales evaluating failures in mentalizing and PSMU fell within the expected range for individuals from the community, suggesting that the severity of failures in mentalizing and PSMU symptoms was not clinically significant for the majority of participants. Females reported a larger amount of time spent on social media and higher levels of preoccupied and fearful attachment styles, failures in mentalizing, and PSMU, whereas males reported higher levels of secure attachment style.

### 3.2. Associations among Variables

Pearson’s *r* correlation coefficients among the study variables are shown in Table 2. Secure attachment style was negatively associated with failures in mentalizing and PSMU, dismissing attachment style was negatively associated with failures in mentalizing, and both preoccupied and fearful attachment styles were positively associated with failures in mentalizing and PSMU. Additionally, sociodemographic characteristics and time spent on social media were significantly correlated with the variables of interest: age was negatively associated with insecure attachment styles (i.e., dismissing, preoccupied, and fearful), failures in mentalizing, and PSMU; years of education were positively associated with a secure attachment style, and negatively associated with preoccupied attachment style, failures in mentalizing, and PSMU; time spent on social media was positively associated with secure attachment style, and negatively associated with both attachment styles characterized by a negative view of the self (i.e., preoccupied and fearful), failures in mentalizing, and PSMU.

The results of multiple linear regression analysis are presented in Table 3. A dismissing attachment style predicted decreased levels of PSMU, whereas adult attachment styles characterized by a negative view of the self (i.e., preoccupied and fearful) and failures in mentalizing predicted increased levels of PSMU. Furthermore, being female, a younger age, and a larger amount of time spent on social media were positive predictors in the model.

**Figure 1:**
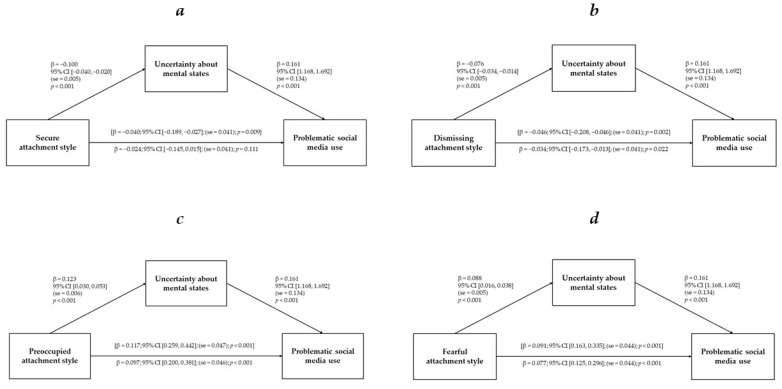
(**a**–**d**). Mediating effects of uncertainty about mental states on the relationships between attachment styles and problematic social media use.

The results of mediation analyses are displayed in Figure 1a–d. We found that failures in mentalizing mediated the relationships between attachment styles and PSMU. In the first model (Figure 1a), the negative association between secure attachment style and PSMU was fully mediated by failures in mentalizing. In the second model (Figure 1b), dismissing attachment style had a negative effect on PSMU through the partial mediating effect of failures in mentalizing. In the third and fourth models, failures in mentalizing partially mediated the positive association between preoccupied attachment style and PSMU (Figure 1c), and between fearful attachment style and PSMU (Figure 1d). Significant associations were found between PSMU and covariates in all models. The sociodemographic characteristics predicting increased levels of PSMU were being female (β = −0.049; 95% CI [−0.829, −0.210]; (se = 0.158); *p* = 0.001) and a younger age (β = −0.132; 95% CI [−0.078, −0.048]; (se = 0.008); *p* < 0.001). Additionally, a larger amount of time spent on social media was associated with increased PSMU (β = 0.347; 95% CI [0.808, 0.960]; (se = 0.039); *p* < 0.001). Finally, secure and insecure attachment styles (i.e., dismissing, preoccupied, and fearful) were significant covariates in all mediation models.

## 4. Discussion

The current study examined the mediating role of failures in mentalizing in the relationship between adult attachment styles and PSMU. Significant sex differences were observed among participants. Females reported higher levels of preoccupied and fearful attachment styles compared to males, who reported higher levels of secure attachment. This finding is consistent with research suggesting that females are more prone to exhibiting anxious attachment attitudes than males [96]. In contrast, higher mentalizing abilities were observed among males. This finding might seem surprising, considering that the relevant literature does not suggest sex differences in mentalization [94,97]; however, it might represent a byproduct of a younger age observed among females in the sample, since a younger age is associated with decreased levels of mentalizing abilities [93]. Additionally, females reported greater levels of PSMU, which aligns with meta-analytic research [83]. According to the literature, a larger amount of time spent on social media, a younger age, and lower education were associated with increased PSMU [78,98,99,100].

Correlation analyses revealed that secure and dismissing attachment styles were negatively associated with failures in mentalizing. This finding suggests that secure and dismissing attachment styles may promote reflective functioning during distressing experiences. In fact, the activation of the attachment system in secure individuals who perceive their efforts to seek proximity and emotional support as effective may sustain reflective functioning [61]; in contrast, the deactivation of the attachment system in individuals with a dismissing attachment style implies the exclusion of disturbing feelings from awareness, preventing failures in cognitive mentalizing [33]. Preoccupied and fearful attachment styles were positively associated with failures in mentalizing. Thus, the hyperactivation of the attachment system in individuals who perceive themselves negatively and experience high levels of anxiety in close relationships may impair mentalizing abilities [61,65].

Secure attachment style was negatively associated with PSMU, whereas preoccupied and fearful attachment styles were positively associated with PSMU. This finding supports previous literature suggesting that secure attachment may reduce the risk of a maladaptive involvement in social media, and that anxious attachment attitudes, which are rooted in a negative view of the self, may foster PSMU [31,47,49,101]. In fact, individuals with preoccupied or fearful attachment styles may excessively resort to social media platforms in order to alleviate anxiety in social interactions and fulfill their needs of relatedness and self-presentation [42,102,103]. In accordance with previous research [71,72], we also found a positive association between failures in mentalizing and PSMU. Accordingly, PSMU may represent an attempt to cope with unprocessed feelings by seeking refuge in an online environment perceived as more predictable and stable than offline interpersonal contexts [73].

Regression analysis showed that attachment styles characterized by a negative view of the self—i.e., preoccupied and fearful attachment styles—predicted increased levels of PSMU. Despite correlation analyses not showing a significant association between dismissing attachment and PSMU, regression analysis displayed that dismissing attachment style had a negative effect on PSMU when this association was controlled for sociodemographic characteristics, time spent on social media, and failures in mentalizing. Accordingly, some individuals with a dismissing attachment style might have a lower risk of engaging in PSMU. Finally, PSMU was predicted by being female, a younger age, and a larger amount of time spent on social media.

Mediation analyses provided further elucidation on the effects of adult attachment styles and failures in mentalizing on PSMU, showing that failures in mentalizing mediated the relationships between adult attachment styles and PSMU. Secure attachment style predicted reduced levels of PSMU, and this relationship was fully mediated by failures in mentalizing. Thus, a secure attachment style may sustain the ability to reflect on one’s own and others’ mental states, promoting adaptive emotion regulation strategies within close relationships [50,51]. As a result, individuals with a secure attachment style might have a lower likelihood of resorting to PSMU [46,86] as a coping strategy for dealing with personal and psychosocial difficulties [8]. Failures in mentalizing partially mediated the negative association between dismissing attachment style and PSMU. Despite previous research showing inconsistent findings on the role of avoidant attachment attitudes in PSMU [31], our findings suggest that individuals who exhibit a dismissing attachment style—characterized by a positive view of the self and the tendency to be self-reliant [36]—and possess proper mentalizing abilities have a low risk of engaging in PSMU. On the one hand, the deactivation of the attachment system, when the levels of distress are not excessively high, may support cognitive mentalizing abilities, preventing the use of social media as an emotion regulation strategy; on the other hand, the tendency to self-reliance may reduce the likelihood of participating in interactions with others through social media. Finally, preoccupied and fearful attachment styles were predictively associated with increased PSMU through the indirect partial effect of failures in mentalizing. These findings extend to previous research showing that the positive relationships between preoccupied attachment style and PSMU, and between fearful attachment style and PSMU, might be explained by different factors, such as the tendency to excessively resort to fantasy [47], low self-esteem [101], the fear of missing out [49], and so on. In fact, anxious attachment attitudes, stemming from a negative view of the self, may foster failures in mentalizing, which in turn may increase the dysfunctional use of social media platforms in order to meet one’s own relational needs and to alleviate negative feelings [16,77].

In line with other literature findings, being female, a younger age, and a larger amount of time spent on social media were significant covariates in all mediation models [78,83,98,99,100]. From a compensatory perspective, the duration of social media usage could be closely associated with the level of psychological vulnerabilities that prompt individuals to engage in such Internet platforms [8].

The current study is not devoid of certain limitations. The sample of the study included individuals from the community, limiting the generalizability of results to individuals who exhibit impairments in their personal and interpersonal functioning due to a maladaptive involvement in social media. Thus, future studies should investigate the role of adult attachment styles and failures in mentalizing in individuals who suffer from high levels of PSMU. Despite the variables of the study being evaluated through well-validated measures, the use of self-report instruments may increase the risk of bias. Accordingly, it is advisable that further research employs structured or semi-structured interviews to assess the variables of interest. Additionally, the cross-sectional design of the study did not allow detection of causal relationships between adult attachment styles, failures in mentalizing, and PSMU. Longitudinal studies are therefore recommended to ascertain the effects of adult attachment styles and failures in mentalizing on PSMU. Additionally, future studies should investigate the role of attachment patterns in various interpersonal contexts, such as relationships with parents, peers, or partners, in preventing or fostering failures in mentalizing and PSMU. Finally, further research is needed to examine the potential moderating or mediating effects of other variables, such as mind-wandering, maladaptive daydreaming, and emotion dysregulation, on the relationships between adult attachment styles and PSMU.

## 5. Conclusions

Its limitations notwithstanding, this study provides evidence for the role of failures in mentalizing in the relationships between adult attachment styles and PSMU. Our findings suggest that secure and dismissing attachment styles may reduce the risk of using social media as a maladaptive coping strategy aimed at dealing with unprocessed feelings; in contrast, anxious attachment attitudes, stemming from a negative view of the self, may increase the likelihood to experience failures in mentalizing abilities, which in turn, increases the vulnerability to PSMU.

Furthermore, the current study can have relevant implications for the assessment and treatment of PSMU. It is advisable that clinicians carefully evaluate the attachment styles and mentalizing abilities of patients who are excessively involved in social media in order to plan tailored interventions. In fact, it is fundamental that clinicians adopt a non-judgmental approach when treating patients with preoccupied or fearful attachment styles, being cautious not to contribute to their feelings of inadequacy in close relationships. Consequently, clinicians should assist patients in establishing a sense of security in therapeutic relationships [33], helping them to reflect on their and others’ mental states and adopt adaptive strategies aimed at managing their negative feelings [104]. Consequently, it might be essential to assist patients in recognizing the role played by their negative representation of the self and high anxiety levels in the adoption of maladaptive self-regulation strategies, such as PSMU. Pursuing these objectives might be essential for mitigating the inclination towards maladaptive utilization of social media and enhancing the individual and interpersonal functioning of people suffering from PSMU.

## Figures and Tables

**Table 1:** Descriptive statistics and sex differences ^1^.

Variable	Full Sample (N = 3600)			Females (N = 2312)		Males (N = 1288)			
	M	(SD)	Range	M	(SD)	M	(SD)	*t*_(3598)_	*p*
Age	29.92	(10.68)	18–60	29.19	(10.34)	31.25	(11.15)	−5.56	<0.01
Years of education	14.75	(2.70)	5–21	14.97	(2.57)	14.37	(2.87)	6.40	<0.01
Time spent on social media (hours)	2.66	(1.99)	0–20	2.80	(2.03)	2.41	(1.88)	5.73	<0.01
RQ—Secure attachment style	3.31	(1.89)	1–7	3.23	(1.89)	3.46	(1.88)	−3.53	<0.01
RQ—Dismissing attachment style	3.13	(1.84)	1–7	3.11	(1.83)	3.16	(1.85)	−0.76	0.45
RQ—Preoccupied attachment style	2.52	(1.69)	1–7	2.56	(1.69)	2.44	(1.68)	2.10	0.04
RQ—Fearful attachment style	3.04	(1.86)	1–7	3.18	(1.89)	2.79	(1.79)	6.15	<0.01
RFQ—Uncertainty about mental states	0.67	(0.57)	0–3	0.69	(0.58)	0.62	(0.55)	3.62	<0.01
BSMAS—Problematic social media use	13.08	(5.07)	6–30	13.50	(5.03)	12.33	(5.04)	6.64	<0.01

^1^ Sex: “Female” was coded as 0, and “Male” was coded as 1; RQ = Relationship Questionnaire, RFQ = Reflective Functioning Questionnaire, and BSMAS = Bergen Social Media Addiction Scale.

**Table 2:** Pearson’s *r* correlations among the investigated variables ^2^.

Variable	2.	3.	4.	5.	6.	7.	8.	9.
1. Age	0.12 **	−0.24 **	0.02	−0.09 **	−0.19 **	−0.25 **	−0.04 *	−0.26 **
2. Years of education	-	−0.09 **	0.09 **	0.02	−0.06 **	−0.03	−0.11 **	−0.06 **
3. Time spent on social media (hours)		-	−0.04 *	−0.01	0.11 **	0.08 **	0.14 **	0.41 **
4. RQ—Secure attachment style			-	−0.14 **	−0.15 **	−0.25 **	−0.14 **	−0.10 **
5. RQ—Dismissing attachment style				-	0.00	0.13 **	−0.06 **	−0.02
6. RQ—Preoccupied attachment style					-	0.29 **	0.18 **	0.21 **
7. RQ—Fearful attachment style						-	0.14 **	0.20 **
8. RFQ—Uncertainty about mental states							-	0.25 **
9. BSMAS—Problematic social media use								-

^2^ RQ = Relationship Questionnaire, RFQ = Reflective Functioning Questionnaire, BSMAS = Bergen Social Media Addiction Scale, * *p* < 0.05, and ** *p* < 0.01.

**Table 3:** Regression model predicting the severity of problematic social media use ^3^.

Variable	β	SE	Partial *r*	*t*	*p*
Sex	−0.04	0.16	−0.05	−2.87	<0.01
Age	−0.14	0.01	−0.15	−8.90	<0.01
Years of education	0.01	0.03	0.01	0.57	0.57
Time spent on social media (hours)	0.33	0.04	0.34	21.78	<0.01
RQ—Secure attachment style	−0.02	0.04	−0.03	−1.59	0.11
RQ—Dismissing attachment style	−0.03	0.04	−0.04	−2.29	0.02
RQ—Preoccupied attachment style	0.10	0.05	0.10	6.28	<0.01
RQ—Fearful attachment style	0.08	0.04	0.08	4.84	<0.01
RFQ—Uncertainty about mental states	0.16	0.13	0.18	10.70	<0.01

^3^ Sex: “Female” was coded as 0, and “Male” was coded as 1; RQ = Relationship Questionnaire, RFQ = Reflective Functioning Questionnaire, and BSMAS = Bergen Social Media Addiction Scale; Model: F (9,3590) = 135.05, *p* < 0.001, and *R*^2^ = 0.25.

## Data Availability

The data presented in this study are available on request. The data are not publicly available due to GDPR 2016/79.
